# Capsaicin sensitivity in patients with chronic cough– results from a cross-sectional study

**DOI:** 10.1186/1745-9974-9-5

**Published:** 2013-02-28

**Authors:** Ewa Ternesten-Hasséus, Christel Larsson, Sven Larsson, Eva Millqvist

**Affiliations:** 1 Department of Allergology, Institution of Internal Medicine, the Sahlgrenska Academy at University of Gothenburg, Gothenburg, Sweden; 2 Department of Otorhinolaryngology, Central Hospital, Skövde, Sweden; 3 Department of Respiratory Medicine, Institution of Internal Medicine, the Sahlgrenska Academy at University of Gothenburg, Gothenburg, Sweden

**Keywords:** Cough, Chemical sensitivity, Capsaicin, Sensory hyperreactivity

## Abstract

**Background:**

A subgroup of patients with chronic cough is recognised as having airway symptoms resulting exposure to chemicals and scents related to enhanced cough sensitivity to inhaled capsaicin. Sensory hyperreactivity, which has an estimated prevalence of more than 6%, is one possible explanation for the symptoms experienced by these patients. We hypothesized that a number of patients diagnosed with chronic unexplained cough also have coughing provoked by chemical irritants associated with augmented capsaicin cough reaction, but the extent of such a relation is not known. One aim of the present study was to analyse cough sensitivity to inhaled capsaicin in patients with chronic unexplained cough. Another aim was to compare capsaicin sensitivity in individuals with chemically induced coughing (the chemical-sensitive group) to capsaicin sensitivity in those without such chemical sensitivity (non-sensitive group).

**Methods:**

Fifty-six participants from an earlier cross-sectional study of 62 patients with chronic unexplained cough were asked to participate in this study: 33 were chemical-sensitive and 23 were non-sensitive. Each participant visited the clinic once and performed a capsaicin inhalation test with one of two inhalation devices. The number of coughs, induced airway symptoms, and spirometry results were recorded.

**Results:**

Thirty-nine of the invited patients participated in the study, with 32 in the chemical-sensitive group (21 women, 11 men), and 7 in the non-sensitive group (4 women, 3 men). The chemical-sensitive patients coughed significantly more on inhaling capsaicin, and had significantly more other airway symptoms compared to those in the non-sensitive group. Women coughed significantly more than men after receiving the higher concentration of capsaicin.

**Conclusions:**

Environmental irritants often trigger chronic unexplained cough. The current findings confirm that this sensitivity is related to enhanced capsaicin cough sensitivity and indicates more involvement of airway sensory nerves in the pathophysiology of the disease than in cough without evident trigger factors.

## Background

About 10-38% of patients seeking medical care in respiratory clinics report coughing as a symptom [1,2]. Individuals suffering from cough, show a high level of morbidity and a high rate of healthcare utilization, and cough has a negative impact on patients’ health-related quality of life [3-6]. Cough is regarded as chronic when it persists for more than 8 weeks [7]. Common causes of chronic cough include asthma, gastroesophageal reflux disease (GERD), and post-nasal drip syndrome [8]. After all possible underlying causes of cough have been excluded and the patients have been treated according to current guidelines, a group of patients, mainly female, remains that can be labelled as having chronic refractory unexplained cough. This cough is often triggered by talking, laughing, singing, or strong chemicals and scents from perfume, cigarette smoke, and cooking [9,10]. More knowledge is needed about cough induced by such irritants.

Sensory hyperreactivity (SHR) is one explanation for cough and other airway symptoms induced by exposure to chemicals and scents [11,12]. However, the extent to which SHR could help explain the symptoms experienced by patients with chronic unexplained cough is not well known. Common symptoms of SHR are cough, heavy breathing, difficulty getting air, phlegm, throat irritation, hoarseness, rhinorrhoea, and eye irritation [11-13]. SHR is most common among women (70%), and a Swedish population-based study estimated its prevalence to be more than 6% among adults [14]. Patients with SHR can be identified through a capsaicin provocation test. Several studies have shown this test has good short-term and long-term reproducibility and the ability to distinguish between patients with SHR and those with asthma and those who are healthy controls [11-13,15].

Capsaicin, the main pungent ingredient in chilli, is a well-known cough-inducing agent when inhaled [16-19]. It is a noxious and odourless vanilloid, which stimulates the unmyelinated C-fibres of the sensory nervous system and produces a burning sensation by activating the ion channel transient receptor vanilloid subunit 1 (TRPV1) [20]. The TRPV1 channel is activated not only by capsaicin, but also by noxious stimuli and heat; it is potentiated by extracellular acidic pH, and interacts with vanilloid compounds [21]. Patients with chronic cough have increased expression of TRPV1 and a significant correlation between capsaicin response and the number of TRPV1-positive nerves [22]. A heightened cough sensitivity to inhaled capsaicin has been found in patients with chronic cough, in cases where the cause of the cough is known and where it is unknown [23].

We hypothesized that a number of patients diagnosed with chronic unexplained cough also have coughing provoked by chemical irritants associated with augmented capsaicin cough reaction but the extent of such a relation is not known. To further explore this, a capsaicin inhalation test could be applied. One aim of the present study was to analyse cough sensitivity to inhaled capsaicin in patients with chronic unexplained cough. The patients had been selected from an earlier cross-sectional study [6]. Another aim was to compare capsaicin sensitivity in individuals with chemically induced airway symptoms (the chemical-sensitive group) to capsaicin sensitivity in those without such chemical sensitivity (non-sensitive group).

## Methods

### Patients

Fifty-six patients from an earlier cross-sectional study that included 62 patients with chronic unexplained cough were randomly selected and invited to participate in this study [6]. The group included 33 chemical-sensitive patients and all of the 23 non-sensitive individuals. The earlier study had already excluded all patients with diseases such as asthma, chronic obstructive pulmonary disease and pulmonary fibrosis that could cause cough and all patients diagnosed with allergy, rhinitis, post-nasal drip syndrome, or any kind of GERD, those making any use of angiotensin-converting enzyme inhibitors or medication for GERD, and current smokers. This study excluded anyone who was pregnant or breastfeeding.

### Study design

The participants were contacted by phone. Informed consent was obtained from all participants after they were provided with verbal and written information. The study was approved by the Regional Ethical Review Board of Gothenburg, Sweden.

The participants visited the clinic once and all of them were again screened using a local questionnaire that asked whether they had airway symptoms from chemicals and scents (yes/no) [6]. None of the patient had performed a capsaicin inhalation test before they were included in the study. Provocations were not performed in patients who had experienced respiratory infections during the past month. Before the capsaicin provocations, all medication was withheld for at least 6 h.

The capsaicin challenges were performed with a standardized method [11-15] with one of two different air-driven devices: Pari Boy (Paul Ritzau Pari-Werk, GmbH, Starnberg, Germany) or Maxin MA3 (Clinova Medical AB, Malmö, Sweden). The two different inhalation devices were used because the allergy clinic switched from using the Pari Boy to using the Maxin MA3 over the period of studies. These devices are compatible in terms of the limits set for a positive capsaicin inhalation test, but use different capsaicin concentration levels [15].

In each challenge the participant inhaled saline followed by two concentrations of capsaicin, the second being stronger than the first. The order of the given concentrations was known by the nurse performing the tests but the patients were not told that the capsaicin concentrations would increase during the provocations. The total time for each provocation was about 35 minutes.

In accordance with the earlier established limits for capsaicin cough sensitivity [12,14], the cut-off values for a positive capsaicin inhalation test for the diagnosis of SHR was set to 10 coughs at the lower capsaicin concentrations (0.4 and 0.06 μmol/L) or 35 coughs at the higher capsaicin concentrations (2.0 and 0.3 μmol/L), with both the devices [12,14,15].

### Capsaicin provocation

A stock solution of capsaicin (1 mmol/L in ethanol [99.5%] from Sigma-Aldrich Sweden AB, Stockholm, Sweden) was prepared. From this stock solution, aqueous provocation solutions were prepared in accordance with earlier studies; the solutions were 0.4 and 2.0 μmol/L for the Pari Boy device and solutions of 0.06 and 0.3 μmol/L for the Maxin MA3 device. The number of coughs was counted manually for 10 min from the start of inhalation of each provocation solution [13,15].

In the case of the Pari Boy device, the device was filled first with 1 mL of saline and then two capsaicin concentrations [11-13,15]. The participants were instructed to inhale with tidal volume breathing without a nose-clip, to completion or for a maximum of 6 min, following by a 4 min rest.

The Maxin MA3 device was filled first with 2 mL saline and then the two capsaicin concentrations. The device nebulizes continually, and provides a fixed constant flow of 0.25 mL/min. The participants were instructed to inhale with tidal volume breathing without a nose-clip for 4 min, followed by 6 min rest, consequently they inhaled a total of 1 mL provocation solution [15].

Before and after the capsaicin provocation, the participants evaluated their symptoms on a scale of 0–3 (0, no symptoms; 1, mild symptoms; 2, moderate symptoms; and 3, severe symptoms). Eight symptoms were analysed: heavy breathing, difficulty getting air, chest pressure, phlegm, throat irritation, hoarseness, rhinorrhoea, and eye irritation [11,24,25].

Forced expiratory volume during 1 s (FEV_1_) was measured before and after each capsaicin provocation (Vitalograph, Buckingham, UK), and the highest of two values was recorded.

### Statistical methods

All data were analysed using version 17 of the SPSS software package (SPSS, Inc., Chicago, IL, USA). Data are presented as mean values with standard deviation (SD) and median values. Results were considered significant at a *p* value of < 0.05. The Mann–Whitney *U*-test was used for non-paired data, and the Wilcoxon signed-rank test for paired data.

In accordance with dose-response relationships seen in earlier studies, missing data for participants whose provocation was halted due to excess coughing were filled in by doubling the number of coughs evoked by the lower capsaicin concentrations to represent the cough response to the higher capsaicin concentrations [11,13,15].

## Results

Of the 56 patients invited to participate, 17 were excluded due to recovery from their coughing: 2 were in the chemical-sensitive group (n = 33) and 11 were in the non-sensitive group (n = 23). In the non-sensitive group, 3 others were excluded because of difficulty in taking time away from work, and 1 because of azithromycin treatment for airway symptoms. All of the chemical-sensitive patients reported persistent sensitivity and one of the previously non-sensitive patients reported symptoms from chemicals and scents and was reassigned to the chemical-sensitive group.

The final analyses was done on the data from 39 patients, 32 (mean age 54.7 [10.8]) in the chemical-sensitive group (21 women and 11 men), and 7 (mean age 49.4 [19.5]) in the non-sensitive group (4 women and 3 men). The demographic data of the study group are shown in Table 1. All patients except for two had had a negative methacholine inhalation test within the last five years, in accordance with international guidelines [26,27]. Of the 39 patients, 5 regularly used inhaled corticosteroids, 4 inhaled β_2_-agonists, 1 inhaled anticholinergic, and 8 used morphine derivate syrup.

**Table 1:** Demographic data for 39 patients with chronic cough

**Characteristics**	**Patients (n = 39)**
Sex, female/male (n)	25/14
Chemical-sensitive/non-sensitive (n)	32/7
Age, years	53.8 (12.6)
Duration of cough symptoms, years	9.4 (6.2)
**Smoking status (n*)***	
Never/previous	24/15

Data are presented as mean and standard deviation (SD) for age and duration of cough symptoms, and otherwise as number (n).

### Capsaicin provocation

Nine patients in the chemical-sensitive group had undergone the capsaicin inhalation test four years earlier with the Pari Boy device [11-13,15], and that data were used in this study. The remaining 30 patients (23 from the chemical-sensitive group, and 7 from the non-sensitive group) were tested using the Maxin MA3 device [15].

**Figure 1:**
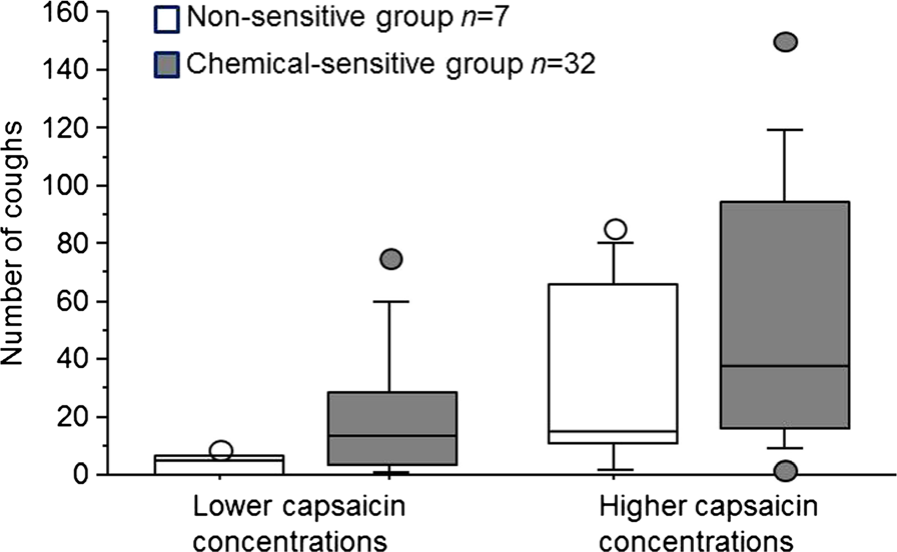
**Box plot presentation of cough response in 39 patients with chronic cough after inhalation of lower (0.4 or 0.06 μmol/L) and higher (2.0 or 0.3 μmol/L) capsaicin concentrations.** The horizontal line in the centre of each box is the median. The top and bottom of the box represent the 25th and 75th percentiles, and whiskers indicate the 10th and 90th percentiles. Circles show individual maximum and minimum data points.

All 39 patients tried to inhale the lower concentrations of capsaicin (0.4 and 0.06 μmol/L for the Pari Boy device and the Maxin MA3 device respectively); 5 patients in the chemical-sensitive group discontinued the provocation because of having more than 35 coughs. All patients in the non-sensitive group inhaled both the low and the high capsaicin concentrations. The chemical-sensitive group coughed significantly more than the non-sensitive group on the lower concentrations of capsaicin (*p* < 0.05). In the chemical-sensitive group, the median number of coughs was 13 for the lower concentrations (0.4 or 0.06 μmol/L) and 37.5 for the higher concentrations (2.0 or 0.3 μmol/L). The corresponding values for the non-sensitive group were 5 and 15, respectively. The results of the capsaicin provocations are presented in Figure 1.

Twenty two (69%) of the patients in the chemical-sensitive group had a positive capsaicin inhalation test, and met the diagnostic criteria for SHR [12,14]. In the non-sensitive group, 3 of 7 patients (43%) had a positive capsaicin inhalation test.

Women coughed significantly more than men on inhaling the higher concentrations of capsaicin (*p* < 0.05). For women the median number of coughs was 10 at the lower concentrations of capsaicin and 55 at the higher concentrations. The corresponding values for men were 6.5 and 23.5 respectively.

The capsaicin provocation also induced significantly greater symptoms in the chemical-sensitive group for heavy breathing (*p* < 0.001), difficulty getting air (*p* < 0.01), chest pressure (*p* < 0.05), phlegm (*p* < 0.05), throat irritation (*p* < 0.01), hoarseness (*p* < 0.01), rhinorrhoea (*p* < 0.001), and eye irritation (*p* < 0.05). In the non-sensitive group, the capsaicin provocation induced significantly more (*p* < 0.05) throat irritation.

The basic mean FEV_1_ was 102.4% (18.4) of predicted value in the chemical-sensitive group and 103.8% (14.9) of predicted value in the non-sensitive group (*ns*), and did not change after any capsaicin provocation.

## Discussion

The main finding of this study was that environmental irritants are often triggering factors in chronic unexplained cough and cough sensitivity to inhaled capsaicin was higher in chemical-sensitive patients than in non-sensitive patients. We also found that 22 (69%) of the patients in the chemical-sensitive group had a positive capsaicin inhalation test, and met the diagnostic criteria for SHR [12,14]. In the non-sensitive group, only 3 of 7 patients (43%) had a positive capsaicin inhalation test. Further, in comparison to previous studies the results indicate higher capsaicin sensitivity in the non-sensitive group than in earlier tested healthy controls [28,29], but the present non-sensitive group is too small to draw any major conclusions. The study included twice as many women as men, and the study results are in accordance with previous reports of women being over-represented in cough clinics [9] and being more sensitive to inhaled capsaicin [30,31].

A total of 13 patients had been excluded from participating because they had recovered from their cough. Eleven belonged to the non-sensitive group, constituting 48% of the non-sensitive individuals. This indicates a possibility of recognising a group of cough patients who have symptoms induced by environmental irritants and who are at risk of having the problems last for several years. However, although increasing evidence suggests environmental irritants are important factors in chronic cough [10,32-34], larger groups of patients need to be studied to find out whether chemical sensitivity is essential for long-lasting symptoms to occur.

The use of two devices, the Pari Boy and Maxin MA3 could be perceived as a limitation. However, an earlier study showed that the Pari Boy and the Maxin MA3 device, which we used in the present study, can be used interchangeably to estimate levels of neural sensory reactivity, and there was good agreement between the cough results of capsaicin with the two devices [15]. Each provocation method had also a good ability to distinguish patients with SHR from healthy controls, although the Maxin MA3 device showed even higher degree of discriminative ability between patients with SHR and healthy control [11,15].

The capsaicin inhalation test is non-specific because of the huge variation in capsaicin cough sensitivity among healthy individuals and in the different conditions affecting the airways [18,35]. The patients in the present study were carefully examined and other causes of cough were excluded. To avoid bias, international guidelines recommend capsaicin concentrations be given randomly, and that saline be randomly interspersed between incremental concentrations of capsaicin [36]. Previous studies have, however, shown that the order in which capsaicin concentrations are given is of importance for the cough outcome of capsaicin provocations [14,18] and that in patients with SHR even inhalation of saline induced coughing [11,37]; these findings influenced our choice of method, with first saline and then low and high concentration of capsaicin being given. Furthermore the participants had not previously been tested with capsaicin and they were not told that the capsaicin concentrations would increase during the provocation. In our experience, the capsaicin inhalation test used herein with tidal breathing is a stable and reproducible method, and represents an objective test to measure sensory reactivity in the airways. The limits used for a positive capsaicin inhalation test were in accordance with those used previous studies in patients with SHR [12,14].

Researchers have sharply disagreed on the cause of chronic unexplained cough [38,39], which included post-nasal drip syndrome [1] and GERD [40,41]. During the last decade, however, a more common view seems to have developed [42] and the post-nasal drip syndrome is now often replaced with a more general description, ‘upper airway cough syndrome’ [43]. The upper and lower airways are viewed as being closely related and complementing each other with regard to reflexes [44-46]. This close connection is also evident in regard to chronic cough. The cough hypersensitivity syndrome is a new paradigm that accounts for unexplained cough and includes several groups of chronic cough patients, both those with symptoms that may indicate a reflux disease and those with a general hypersensitivity towards, for example, environmental irritants [32,33,47]. The current study demonstrates that an association exists between the upper airways and chronic cough, because many patients also had rhinitis symptoms after the capsaicin provocations. It also shows a general airway hypersensitivity linked to increased capsaicin cough sensitivity.

## Conclusions

Environmental irritants often trigger chronic unexplained cough. The current findings confirm that this sensitivity is related to enhanced capsaicin cough sensitivity and indicates more involvement of airway sensory nerves in the pathophysiology of the disease than in cough without evident trigger factors.

## Abbreviations

FEV_1_: Forced expiratory volume during one second; GERD: Gastroesophageal reflux disease; Ns: Not significant; SD: Standard deviation; SHR: Sensory hyperreactivity; TRPV1: Transient receptor potential vanilloid type 1.

## Competing interests

The authors declare that they have no competing interests.

## Authors’ contributions

All the authors participated in the design of the study. ETH coordinated and analysed the data and drafted the manuscript. EM analysed the data and drafted the manuscript. CL and SL helped to draft the manuscript. All authors have read and approved the final manuscript.
